# Supplementary material for: The changing career paths of PhDs and postdocs trained at EMBL
Source: eLife. 2023 Nov 23;12:e78706. doi: 10.7554/eLife.78706 (PMC10666930; doi:10.7554/eLife.78706)
Supplement: Supplementary file 1. [file elife-78706-supp1.docx]

**Lu et al. 2023. The changing career paths of PhDs and postdocs trained at EMBL. *eLife* 12:e78706**

### Supplementary File 1: Supplementary tables

### Table S1: Descriptive statistics for the key data set used for figures

| **Variable** | **Stats / Values** | **Freqs (% of Valid)** | **Valid** | **Missing** | **Description** |
| --- | --- | --- | --- | --- | --- |
| unique_ID  [character] | [ 2284 unique values ] |  | 2284  (100.0%) | 0  (0.0%) | unique ID number (F + unique random number) |
| type_pre_postdoc  [character] | 1. postdoc  2. predoc | 1315 (57.6%)   969 (42.4%) | 2284  (100.0%) | 0  (0.0%) | predoc (PhD student) or postdoc |
| gender  [character] | 1. f  2. m | 907 (39.7%)  1377 (60.3%) | 2284  (100.0%) | 0  (0.0%) | (f= female, m=male) |
| nationality  [character] | 1. [coding suppressed]  2. [coding suppressed]  3. [coding suppressed]  4. [coding suppressed]  5. [coding suppressed]  6. [coding suppressed]  7. [coding suppressed]  8. [coding suppressed]  9. [coding suppressed]  10. [coding suppressed]  [ 82 others ] | 486 (21.3%)  239 (10.5%)  206 ( 9.0%)  152 ( 6.7%)  121 ( 5.3%)   95 ( 4.2%)   66 ( 2.9%)   59 ( 2.6%)   58 ( 2.5%)   56 ( 2.5%)  746 (32.7%) | 2284  (100.0%) | 0  (0.0%) | Pseudomized Nationality of ECR |
| phd_year_if_known  [integer] | Mean (sd) : 2007.6 (7.1)  min < med < max:  1984 < 2008 < 2020  IQR (CV) : 11 (0) | 36 distinct values | 1870  (81.9%) | 414  (18.1%) | Year PhD awarded (if known) |
| from.year  [integer] | Mean (sd) : 2005.8 (6.8)  min < med < max:  1986 < 2007 < 2019  IQR (CV) : 12 (0) | 33 distinct values | 2284  (100.0%) | 0  (0.0%) | Start year as PhD student or postdoc |
| to_year  [integer] | Mean (sd) : 2009.4 (6.8)  min < med < max:  1997 < 2010 < 2020  IQR (CV) : 11 (0) | 24 distinct values | 2284  (100.0%) | 0  (0.0%) | Year of PhD defence or end of postdoc contract |
| cohort  [character] | 1. 1997-2004  2. 2005-2012  3. 2013-2020 | 625 (27.4%)  763 (33.4%)  896 (39.2%) | 2284  (100.0%) | 0  (0.0%) | Cohorts based  on to_year (year of PhD defense or year they left the EMBL Postdoc Programme) |
| completeness  [character] | 1. Complete CV  2. Current only  3. No info  4. Partial CV | 1626 (71.2%)   274 (12.0%)   175 ( 7.7%)   209 ( 9.2%) | 2284  (100.0%) | 0  (0.0%) | Complete CV is for those who we could trace a detailed career path for,  with max. two 1-year career breaks. |
| position_1  [character] | 1. AcFac  2. AcOt  3. AcPD  4. IndR  5. laterPI  6. NonRes  7. NonSci  8. unknown | 198 ( 8.7%)  280 (12.3%)  827 (36.2%)  217 ( 9.5%)  113 ( 4.9%)  146 ( 6.4%)   56 ( 2.5%)  447 (19.6%) | 2284  (100.0%) | 0  (0.0%) | Type of role they had at particular timepoints: last (position in 2021) and positions at 1,3,5  etc years after pre/postdoc contract ends)).  8 classifications were used: ·   AcFac [principal investigator in academia] ·   AcOt [other academic research / teaching role] ·   AcPD [academic postdoc] ·   IndR [Industry Research] ·   NonRes [science related non-research professions] ·   NonSci [non scientific professions]  ·   Later PI [position unknown at this timepoint, but became PI]  · unknown  The cell is empty if the ECR has not yet reached this timepoint |
| position_5  [character] | 1. (Empty string)  2. AcFac  3. AcOt  4. AcPD  5. IndR  6. laterPI  7. NonRes  8. NonSci  9. unknown | 431 (18.9%)  350 (15.3%)  239 (10.5%)  357 (15.6%)  223 ( 9.8%)  106 ( 4.6%)  187 ( 8.2%)   60 ( 2.6%)  331 (14.5%) | 2284  (100.0%) | 0  (0.0%) |  |
| position_9  [character] | 1. (Empty string)  2. AcFac  3. AcOt  4. AcPD  5. IndR  6. laterPI  7. NonRes  8. NonSci  9. unknown | 896 (39.2%)  397 (17.4%)  180 ( 7.9%)   74 ( 3.2%)  165 ( 7.2%)   81 ( 3.5%)  183 ( 8.0%)   43 ( 1.9%)  265 (11.6%) | 2284  (100.0%) | 0  (0.0%) |  |
| position_13  [character] | 1. (Empty string)  2. AcFac  3. AcOt  4. AcPD  5. IndR  6. laterPI  7. NonRes  8. NonSci  9. unknown | 1287 (56.3%)   332 (14.5%)    94 ( 4.1%)    19 ( 0.8%)   107 ( 4.7%)    69 ( 3.0%)   150 ( 6.6%)    31 ( 1.4%)   195 ( 8.5%) | 2284  (100.0%) | 0  (0.0%) |  |
| position_17  [character] | 1. (Empty string)  2. AcFac  3. AcOt  4. AcPD  5. IndR  6. laterPI  7. NonRes  8. NonSci  9. unknown | 1659 (72.6%)   232 (10.2%)    57 ( 2.5%)     5 ( 0.2%)    68 ( 3.0%)    48 ( 2.1%)    78 ( 3.4%)    22 ( 1.0%)   115 ( 5.0%) | 2284  (100.0%) | 0  (0.0%) |  |
| timepointLast  [character] | 1. AcFac  2. AcOt  3. AcPD  4. IndR  5. NonRes  6. NonSci  7. unknown | 636 (27.8%)  383 (16.8%)  244 (10.7%)  332 (14.5%)  349 (15.3%)   91 ( 4.0%)  249 (10.9%) | 2284  (100.0%) | 0  (0.0%) |  |
| was_GL  [character] | 1. (Empty string)  2. y | 1599 (70.0%)   685 (30.0%) | 2284  (100.0%) | 0  (0.0%) | y, if ECR held at least one “AcFac” position |
| EMBLtoPI  [integer] | Mean (sd) : 3.8 (3.5)  min < med < max:  -1 < 3 < 18  IQR (CV) : 5 (0.9) | 19 distinct values | 539  (23.6%) | 1745  (76.4%) | Number of calendar years between completing their PhD/postdoc at EMBL and the year they started their first principal investigator role (if known). |
| PhDtoGL  [integer] | Mean (sd) : 6.8 (3.3)  min < med < max:  1 < 6 < 21  IQR (CV) : 3 (0.5) | 21 distinct values | 489  (21.4%) | 1795  (78.6%) | Number of calendar years between their PhD and the year they started their first principal investigator role (if known). |
| new_AcFac_previous  [character] | 1. (Empty string)  2. AcOt  3. AcPD  4. AcPhD  5. IndR  6. NonRes  7. NonSci | 1745 (76.4%)   111 ( 4.9%)   406 (17.8%)     6 ( 0.3%)    14 ( 0.6%)     1 ( 0.0%)     1 ( 0.0%) | 2284  (100.0%) | 0  (0.0%) | For alumni who held an ‘AcFac’ position, this cell lists the classification of the position held before the individual’s first ‘AcFac’ role. |
| CV_wasAcOt  [character] | 1. (Empty string)  2. y | 1617 (70.8%)   667 (29.2%) | 2284  (100.0%) | 0  (0.0%) | y, if ECR held at least one “AcOt” position |
| CV_PhDtoAcOt  [integer] | Mean (sd) : 5.6 (3.1)  min < med < max:  -1 < 6 < 17  IQR (CV) : 4 (0.6) | 17 distinct values | 396  (17.3%) | 1888  (82.7%) | Number of calendar years between their PhD completion and the year they started their first AcOt role (if known/applicable). |
| CV_EMBLtoAcOt  [integer] | Mean (sd) : 2.4 (3.1)  min < med < max:  -1 < 1 < 14  IQR (CV) : 4 (1.3) | 16 distinct values | 477  (20.9%) | 1807  (79.1%) | Number of calendar years between completing their PhD/postdoc at EMBL and the year they started their year they started their first AcOt role (if known/applicable) |
| new_AcOt_previous  [character] | 1. (Empty string)  2. AcFac  3. AcPD  4. AcPhD  5. IndR  6. NonRes  7. NonSci | 1807 (79.1%)     7 ( 0.3%)   393 (17.2%)    38 ( 1.7%)    21 ( 0.9%)    12 ( 0.5%)     6 ( 0.3%) | 2284  (100.0%) | 0  (0.0%) | For alumni who held an ‘AcOt’ position, this cell lists the classification of the position held before the individual’s first ‘AcOt’ role. |
| CV_wasIndR  [character] | 1. (Empty string)  2. y | 1811 (79.3%)   473 (20.7%) | 2284  (100.0%) | 0  (0.0%) | y, if ECR held at least one “IndR” position |
| CV_PhDtoIndR  [integer] | Mean (sd) : 5 (4.2)  min < med < max:  -2 < 4 < 19  IQR (CV) : 5 (0.8) | 22 distinct values | 363  (15.9%) | 1921  (84.1%) | Number of calendar years between their PhD completion and the year they started their first IndR role (if known/applicable). |
| CV_EMBLtoIndR  [integer] | Mean (sd) : 2.8 (3.7)  min < med < max:  -2 < 1 < 20  IQR (CV) : 4 (1.3) | 21 distinct values | 414  (18.1%) | 1870  (81.9%) | Number of calendar years between completing their PhD/postdoc at EMBL and the year they started their year they started their first IndR role (if known/applicable |
| new_IndR_previous  [character] | 1. (Empty string)  2. AcFac  3. AcOt  4. AcPD  5. AcPhD  6. NonRes  7. NonSci | 1869 (81.8%)    23 ( 1.0%)    55 ( 2.4%)   234 (10.2%)    84 ( 3.7%)    15 ( 0.7%)     4 ( 0.2%) | 2284  (100.0%) | 0  (0.0%) | For alumni who held an ‘IndR’ position, this cell lists the classification of the position held before the individual’s first ‘IndR’ role. |
| CV_wasNonR  [character] | 1. (Empty string)  2. y | 1857 (81.3%)   427 (18.7%) | 2284  (100.0%) | 0  (0.0%) | y, if ECR held at least one “NonR” position |
| CV_PhDtoNonRes  [integer] | Mean (sd) : 5.3 (4.1)  min < med < max:  -1 < 5 < 22  IQR (CV) : 6 (0.8) | 20 distinct values | 314  (13.7%) | 1970  (86.3%) | Number of calendar years between their PhD completion and the year they started their first NonRes role (if known/applicable). |
| CV_EMBLtoNonRes  [integer] | Mean (sd) : 3.9 (4.2)  min < med < max:  -7 < 2 < 20  IQR (CV) : 5.2 (1.1) | 23 distinct values | 364  (15.9%) | 1920  (84.1%) | Number of calendar years between completing their PhD/postdoc at EMBL and the year they started their year they started their first NonRes role (if known/applicable |
| new_SciR_previous  [character] | 1. (Empty string)  2. AcFac  3. AcOt  4. AcPD  5. AcPhD  6. IndR  7. NonSci | 1920 (84.1%)     8 ( 0.4%)    37 ( 1.6%)   150 ( 6.6%)    70 ( 3.1%)    61 ( 2.7%)    38 ( 1.7%) | 2284  (100.0%) | 0  (0.0%) | y, if ECR held at least one “NonSci” position |
| CV_wasNonSci  [character] | 1. (Empty string)  2. y | 2122 (92.9%)   162 ( 7.1%) | 2284  (100.0%) | 0  (0.0%) | For alumni who held an ‘NonSci’ position, this cell lists the classification of the position held before the individual’s first ‘NonSci’  role. |
| CV_PhDtoNonSci  [integer] | Mean (sd) : 4.2 (4)  min < med < max:  -1 < 3 < 17  IQR (CV) : 6 (1) | 17 distinct values | 117  (5.1%) | 2167  (94.9%) | Number of calendar years between their PhD completion and the year they started their first NonSci role (if known/applicable). |
| CV_EMBLtoNonSci  [integer] | Mean (sd) : 3.2 (3.7)  min < med < max:  -1 < 2 < 17  IQR (CV) : 4 (1.1) | 17 distinct values | 131  (5.7%) | 2153  (94.3%) | Number of calendar years between completing their PhD/postdoc at EMBL and the year they started their year they started their first NonSci role (if known/applicable |
| new_NonSci_previous  [character] | 1. (Empty string)  2. AcFac  3. AcOt  4. AcPD  5. AcPhD  6. IndR  7. NonRes | 2153 (94.3%)     2 ( 0.1%)     8 ( 0.4%)    44 ( 1.9%)    33 ( 1.4%)    13 ( 0.6%)    31 ( 1.4%) | 2284  (100.0%) | 0  (0.0%) | For alumni who held a Non-research science-related ‘NonRes ‘ position, this cell lists the classification of the position held before the individual’s first ‘NonRes’ role. |
| PUBS_ALL_TOTAL  [integer] | Mean (sd) : 4.5 (4.8)  min < med < max:  0 < 3 < 64  IQR (CV) : 4 (1.1) | 37 distinct values | 2284  (100.0%) | 0  (0.0%) | **Total number of publications linked to EMBL in Web of Science that include this person as an author, excluding retracted publications and corrections.** |
| PUBS_ALL_JIF_count  [integer] | Mean (sd) : 4.5 (4.8)  min < med < max:  0 < 3 < 64  IQR (CV) : 5 (1.1) | 37 distinct values | 2284  (100.0%) | 0  (0.0%) | For each ECR, the number of EMBL publications for which a journal impact factor (JIF) is available. |
| PUBS_ALL_JIF_mean  [numeric] | Mean (sd) : 10 (7.4)  min < med < max:  0 < 8.2 < 74.7  IQR (CV) : 8.1 (0.7) | 1758 distinct values | 2043  (89.4%) | 241  (10.6%) | For each ECR, the average JIF  for all of their EMBL publications |
| PUBS_ALL_JIF_SUM  [numeric] | Mean (sd) : 51.6 (62.6)  min < med < max:  0.7 < 31.7 < 685.5  IQR (CV) : 49.7 (1.2) | 1749 distinct values | 2022  (88.5%) | 262  (11.5%) | For each ECR, the sum of the JIFs for all of their EMBL publications |
| pubs_ALL_JIF_MAX  [numeric] | Mean (sd) : 19.8 (15.1)  min < med < max:  0.7 < 12.1 < 74.7  IQR (CV) : 29.8 (0.8) | 222 distinct values | 2022  (88.5%) | 262  (11.5%) | For each ECR, the highest JIF of their EMBL publications |
| pubs_All_pecentile_mean  [numeric] | Mean (sd) : 65.1 (21.8)  min < med < max:  0 < 67.8 < 100  IQR (CV) : 29.8 (0.3) | 2016 distinct values | 2043  (89.4%) | 241  (10.6%) | For each ECR, the mean of the “percentile in subject area” f for all their EMBL publications |
| pubs_All_percentile_MAX  [numeric] | Mean (sd) : 86.4 (20.5)  min < med < max:  0 < 94.8 < 100  IQR (CV) : 15 (0.2) | 1542 distinct values | 2043  (89.4%) | 241  (10.6%) | For each ECR, the highest value of “percentile in subject area” for all their EMBL publications |
| PUBS_ALL_CNCI_mean  [numeric] | Mean (sd) : 2.8 (5.4)  min < med < max:  0 < 1.6 < 88.8  IQR (CV) : 2 (1.9) | 1982 distinct values | 2043  (89.4%) | 241  (10.6%) | For each ECR, the mean of the “category normalized citation impact” for their EMBL publications |
| PUBS_ALL_CNCI_max  [numeric] | Mean (sd) : 9 (28.7)  min < med < max:  0 < 3 < 441.2  IQR (CV) : 5.1 (3.2) | 1537 distinct values | 2043  (89.4%) | 241  (10.6%) | For each ECR, the highest value of “CNCI” for their EMBL publications |
| pubs_All_published.first  [integer] | Mean (sd) : 2.4 (1.6)  min < med < max:  0 < 2 < 14  IQR (CV) : 2 (0.7) | 14 distinct values | 2047  (89.6%) | 237  (10.4%) | Number of calendar years between EMBL start year and the publication date of their earliest publication |
| pubs_All_published.last  [integer] | Mean (sd) : 5.4 (2.4)  min < med < max:  0 < 5 < 18  IQR (CV) : 3 (0.4) | 19 distinct values | 2047  (89.6%) | 237  (10.4%) | For each ECR, the number of calendar years between EMBL start year and the publication date of their most publication |
| pubs_All_published_authors_mean  [numeric] | Mean (sd) : 8.7 (9.5)  min < med < max:  1 < 6.7 < 201  IQR (CV) : 4.2 (1.1) | 470 distinct values | 2047  (89.6%) | 237  (10.4%) | For each ECR, the mean number of authors on their research articles |
| pubs_RAonly_TOTAL  [integer] | Mean (sd) : 3.6 (3.9)  min < med < max:  0 < 3 < 53  IQR (CV) : 4 (1.1) | 31 distinct values | 2284  (100.0%) | 0  (0.0%) | **Total number of research articles linked to EMBL in Web of Science that include this person as an author, excluding retracted publications and corrections.** |
| pubs_RAonly_JIF_count  [integer] | Mean (sd) : 3.6 (3.9)  min < med < max:  0 < 3 < 53  IQR (CV) : 4 (1.1) | 31 distinct values | 2284  (100.0%) | 0  (0.0%) | Number of research articles for which a JIF is available.  . |
| pubs_RAonly_JIF_mean  [numeric] | Mean (sd) : 11 (8.1)  min < med < max:  0 < 8.9 < 74.7  IQR (CV) : 8.9 (0.7) | 1617 distinct values | 1981  (86.7%) | 303  (13.3%) | For each ECR,  the mean of the JIF for their EMBL research articles |
| pubs_RAonly_JIF_SUM  [numeric] | Mean (sd) : 46.3 (55.7)  min < med < max:  1 < 27.9 < 645.7  IQR (CV) : 46.1 (1.2) | 1610 distinct values | 1966  (86.1%) | 318  (13.9%) | For each ECR, the sum of the JIFs for all of their EMBL research articles |
| pubs_RA_JIF_max  [numeric] | Mean (sd) : 19.4 (14.8)  min < med < max:  1 < 12.1 < 74.7  IQR (CV) : 29.8 (0.8) | 192 distinct values | 1966  (86.1%) | 318  (13.9%) | For each ECR, the highest JIF for their EMBL research articles |
| pubs_RAonly_percentile_mean  [numeric] | Mean (sd) : 73.3 (18.9)  min < med < max:  0 < 76.7 < 100  IQR (CV) : 24.6 (0.3) | 1945 distinct values | 1981  (86.7%) | 303  (13.3%) | For each ECR, the mean of the “percentile in subject area” for their EMBL research articles |
| pubs_RA_pecentile_MAX  [numeric] | Mean (sd) : 87.5 (17.8)  min < med < max:  0 < 94.8 < 100  IQR (CV) : 14.6 (0.2) | 1471 distinct values | 1981  (86.7%) | 303  (13.3%) | For each ECR, the highest value of “percentile in subject area” for their EMBL research articles |
| pubs_RA_CNCI_mean  [numeric] | Mean (sd) : 3.1 (5.8)  min < med < max:  0 < 1.8 < 109.3  IQR (CV) : 2.2 (1.9) | 1939 distinct values | 1981  (86.7%) | 303  (13.3%) | For each ECR, the mean of the “category normalized citation impact” for their EMBL research articles |
| pubs_RAonly_CNCI_max  [numeric] | Mean (sd) : 8.1 (24.1)  min < med < max:  0 < 2.9 < 425.9  IQR (CV) : 4.6 (3) | 1482 distinct values | 1981  (86.7%) | 303  (13.3%) | For each ECR, the highest value of “CNCI” for their EMBL research articles |
| pubs_RAonly_publishedfirst  [integer] | Mean (sd) : 2.7 (1.7)  min < med < max:  0 < 2 < 14  IQR (CV) : 3 (0.6) | 14 distinct values | 1985  (86.9%) | 299  (13.1%) | Number of calendar years between EMBL start year and the publication date of their earliest for research article |
| pubs_RAonly_publishedlast  [integer] | Mean (sd) : 5.5 (2.4)  min < med < max:  0 < 5 < 18  IQR (CV) : 3 (0.4) | 19 distinct values | 1985  (86.9%) | 299  (13.1%) | For each ECR, the number of calendar years between EMBL start year and the publication date of their most recent research article |
| pubs_RAonly_Authors_average  [numeric] | Mean (sd) : 9.2 (9.9)  min < med < max:  1 < 7 < 201  IQR (CV) : 5 (1.1) | 404 distinct values | 1985  (86.9%) | 299  (13.1%) | For each ECR, the mean number of authors on their research articles |
| pubs_FIRST_ra_only_TOTAL  [integer] | Mean (sd) : 1.6 (1.7)  min < med < max:  0 < 1 < 19  IQR (CV) : 2 (1.1) | 15 distinct values | 2284  (100.0%) | 0  (0.0%) | **Total number research articles linked to EMBL in Web of Science that include this person as first author or other lead author (co-first or last), excluding retracted publications and corrections** |
| pubs_FIRST_ra_only_JIF_count  [integer] | Mean (sd) : 1.6 (1.7)  min < med < max:  0 < 1 < 19  IQR (CV) : 2 (1.1) | 15 distinct values | 2284  (100.0%) | 0  (0.0%) | Number of first author research articles for which a JIF is available. |
| pubs_FIRST_ra_only_JIF_mean  [numeric] | Mean (sd) : 10.5 (9.4)  min < med < max:  0 < 8 < 64.8  IQR (CV) : 7.6 (0.9) | 966 distinct values | 1658  (72.6%) | 626  (27.4%) | For each ECR,  the mean of the JIF for their first-author EMBL research articles |
| pubs_FIRST_ra_only_JIFsum  [numeric] | Mean (sd) : 21.7 (21.9)  min < med < max:  1 < 13.1 < 165.3  IQR (CV) : 20.8 (1) | 973 distinct values | 1633  (71.5%) | 651  (28.5%) | For each ECR, the sum of the JIFs for all of their EMBL first-author research articles |
| pubs_FIRST_RA_JIF_max  [numeric] | Mean (sd) : 14.6 (13.1)  min < med < max:  1 < 9.9 < 64.8  IQR (CV) : 10.3 (0.9) | 205 distinct values | 1633  (71.5%) | 651  (28.5%) | For each ECR, the highest JIF for their first-author EMBL research articles |
| pubs_FIRST_RA_pecentile_mean  [numeric] | Mean (sd) : 72.3 (21.8)  min < med < max:  0 < 76.8 < 100  IQR (CV) : 30.9 (0.3) | 1597 distinct values | 1658  (72.6%) | 626  (27.4%) | For each ECR, the mean of the “percentile in subject area” for their first-author EMBL research articles |
| pubs_FIRST_RA_pecentile_MAX  [numeric] | Mean (sd) : 81.1 (21.6)  min < med < max:  0 < 89.9 < 100  IQR (CV) : 24.8 (0.3) | 1488 distinct values | 1658  (72.6%) | 626  (27.4%) | For each ECR, the highest value of “percentile in subject area” for their first-author EMBL research articles |
| pubs_FIRST_RA_CNCI_mean  [numeric] | Mean (sd) : 2.6 (4.9)  min < med < max:  0 < 1.5 < 92.7  IQR (CV) : 2 (1.9) | 1578 distinct values | 1658  (72.6%) | 626  (27.4%) | For each ECR, the mean of the “category normalized citation impact” for their first-author EMBL research articles |
| pubs_FIRST_RA_CNCI_max  [numeric] | Mean (sd) : 4 (8.9)  min < med < max:  0 < 2 < 184.2  IQR (CV) : 3.1 (2.2) | 1482 distinct values | 1658  (72.6%) | 626  (27.4%) | For each ECR, the highest value of “CNCI” for their first-author EMBL research articles |
| pubs_FIRST_ra_only_publishedfirst  [integer] | Mean (sd) : 3.3 (1.9)  min < med < max:  0 < 3 < 17  IQR (CV) : 2 (0.6) | 14 distinct values | 1666  (72.9%) | 618  (27.1%) | Number of calendar years between EMBL start year and the publication date of their earliest for research article |
| pubs_FIRST_ra_only_publishedlast  [integer] | Mean (sd) : 4.8 (2)  min < med < max:  0 < 5 < 17  IQR (CV) : 2 (0.4) | 18 distinct values | 1666  (72.9%) | 618  (27.1%) | For each ECR, the number of calendar years between EMBL start year and the publication date of their most recent research article |
| pubs_FIRST_ra_only_Authors_mean  [numeric] | Mean (sd) : 7 (6.4)  min < med < max:  1 < 5.6 < 105.5  IQR (CV) : 4 (0.9) | 153 distinct values | 1666  (72.9%) | 618  (27.1%) | For each ECR, the mean number of authors on their research articles |
| groupleader_seniority  [character] | 1. (Empty string)  2. junior  3. senior | 14 ( 0.6%)  1078 (47.2%)  1192 (52.2%) | 2284  (100.0%) | 0  (0.0%) | Junior (GL not a senior scientist and had spent <10 years at EMBL when ECR left) / senior (senior scientist or ≥10 at EMBL when ECR left) |
| Group_pubs_ALL_TOTALrecords  [integer] | Mean (sd) : 99.3 (118.3)  min < med < max:  0 < 63 < 688  IQR (CV) : 82 (1.2) | 98 distinct values | 2268  (99.3%) | 16  (0.7%) | Total of **pubs_ALL_TOTAL** for all ECRs in this study with this group leader [note: publications with more than one co-author counted multiple times] |
| Group_pubs_ALL_TOTALmean  [numeric] | Mean (sd) : 4.6 (2.3)  min < med < max:  0.5 < 3.9 < 13.3  IQR (CV) : 2.2 (0.5) | 141 distinct values | 2266  (99.2%) | 18  (0.8%) | Average number of WOS indexed publications from ECRs in this study with this person’s PI .- for each group [Group_pubs_ALL_TOTALrecords / [number of ECRs from the research group who were included in this study] |
| Group_pubs_ALL_TOTAL_wJIF  [integer] | Mean (sd) : 99.2 (118.3)  min < med < max:  1 < 63 < 688  IQR (CV) : 82 (1.2) | 98 distinct values | 2266  (99.2%) | 18  (0.8%) | Number of publication records of ECRs in this group for which a JIF is available |
| Group_pubs_ALL_JIFmean  [numeric] | Mean (sd) : 10 (3.8)  min < med < max:  1.5 < 9.9 < 25.2  IQR (CV) : 5 (0.4) | 213 distinct values | 2266  (99.2%) | 18  (0.8%) | Average JIF of papers with an author from this group (Total of pubs_ALL_JIF_SUM for all ECRs with this group leader / **Group_pubs_ALL_TOTAL_wJIF**). Note: papers with multiple authors are included multiple times in the calculation |
| Group_pubs_ALL_JIFtotalmean  [numeric] | Mean (sd) : 45.9 (32.6)  min < med < max:  1.1 < 38.7 < 199.1  IQR (CV) : 29.8 (0.7) | 213 distinct values | 2266  (99.2%) | 18  (0.8%) | Average cumulative JIF for all papers for each ECR in this study with this person’s PI (includes those from this person). [Total of pubs_ALL_JIF_SUM for all ECRs with this group leader / [number of ECRs from the research group who were included in this study]] Note: papers with two co-first authors are included twice in the calculation |
| Group_pubs_FIRST_ra_only_TOTALrecords  [integer] | Mean (sd) : 34.3 (37.2)  min < med < max:  0 < 23 < 209  IQR (CV) : 32 (1.1) | 54 distinct values | 2266  (99.2%) | 18  (0.8%) | Total of **pubs_FIRST_ra_only_TOTAL** for all ECRs with this group leader [note: publications with multiple co-first authors counted multiple times] |
| Group_pubsFIRST_ra_only_TOTALmean  [numeric] | Mean (sd) : 1.6 (0.7)  min < med < max:  0 < 1.4 < 6  IQR (CV) : 0.9 (0.4) | 107 distinct values | 2266  (99.2%) | 18  (0.8%) | Average number of first author research articles from ECRs in this study with this person’s PI (includes those from this person) [**Group_pubs_FIRST_ra_only_TOTALrecords** / [number of ECRs from the research group who were included in this study]] |
| Group_pubs_FIRST_ra_only_TOTALrecords_wJIF  [integer] | Mean (sd) : 34.4 (37.4)  min < med < max:  1 < 23 < 210  IQR (CV) : 32 (1.1) | 53 distinct values | 2259  (98.9%) | 25  (1.1%) | Total number of publication records for all ECRs with this group leader that have a JIF [note: publications with multiple co-firsts counted multiple times] |
| Group_pubsFIRST_RAonly_JIFmean  [numeric] | Mean (sd) : 10.2 (4.2)  min < med < max:  1.4 < 9.8 < 25.7  IQR (CV) : 6 (0.4) | 205 distinct values | 2259  (98.9%) | 25  (1.1%) | Average JIF of research artilces with a first author from this group (Total of pubs_FIRST_ra_only_JIF_SUM for all ECRs with this group leader, divided by **Group_pubs_FIRST_ra_only_TOTALrecords**). Note: papers with two co-first authors are included twice in the calculation |
| Group_pubsFIRST_ra_only_JIFtotalmean  [numeric] | Mean (sd) : 15.6 (8.4)  min < med < max:  0 < 15.1 < 49.8  IQR (CV) : 10.6 (0.5) | 208 distinct values | 2266  (99.2%) | 18  (0.8%) | Average cumulative JIF for first author research articles for each ECR in this study with this person’s PI (includes those from this person). [Total of pubs_FIRST_ra_only_JIF_SUM for all ECRs with this group leader / [number of ECRs from the research group who were included in this study]] Note: papers with two co-first authors are included twice in the calculation |
| Group_pubs_FIRST_ra_only_UNIQUE_pubs  [integer] | Mean (sd) : 31.9 (33.8)  min < med < max:  1 < 22 < 189  IQR (CV) : 32 (1.1) | 50 distinct values | 2259  (98.9%) | 25  (1.1%) | Number of unique research articles with an ECR from our study in this group as a first author (papers with 2 ifrst authors from same group counted once) |
| Group_pubs_FIRST_ra_only_UNIQUE_pubs_wJIF  [integer] | Mean (sd) : 31.8 (33.8)  min < med < max:  1 < 22 < 189  IQR (CV) : 32 (1.1) | 48 distinct values | 2259  (98.9%) | 25  (1.1%) | Number of unique research articles with an ECR from our study in this group as a first author that have a JIF |
| Group_pubs_FIRST_ra_only_JIF_mean_alternative  [numeric] | Mean (sd) : 10.1 (4.2)  min < med < max:  1.4 < 9.5 < 28.5  IQR (CV) : 5.6 (0.4) | 205 distinct values | 2259  (98.9%) | 25  (1.1%) | Average JIF for the papers in Group_pubs_FIRST_ra_only_UNIQUE_pubs Note: papers with two co-first authors are included ONCE in the calculation |
| Group_pubs_FIRST_ra_only_AUTHORmean  [numeric] | Mean (sd) : 6.7 (3.2)  min < med < max:  2 < 6 < 47  IQR (CV) : 2.5 (0.5) | 153 distinct values | 2259  (98.9%) | 25  (1.1%) | Average number of authors (including those outside this study) on the papers in Group_pubs_FIRST_ra_only_UNIQUE_pubs |
| Group_pubs_FIRST_ra_only_CNCImean  [numeric] | Mean (sd) : 2.5 (2.1)  min < med < max:  0 < 2.1 < 26.7  IQR (CV) : 1.7 (0.8) | 206 distinct values | 2259  (98.9%) | 25  (1.1%) | Average CNCI for papers on the papers in Group_pubs_FIRST_ra_only_UNIQUE_pubs  Note: papers with two co-first authors from this group are included ONCE in the calculation |
| Group_pubs_FIRST_ra_only_PERCENTILEmean  [numeric] | Mean (sd) : 71.7 (10.5)  min < med < max:  0 < 72.8 < 99.5  IQR (CV) : 13 (0.1) | 206 distinct values | 2259  (98.9%) | 25  (1.1%) | Average percentile in subject area on the papers in Group_pubs_FIRST_ra_only_UNIQUE_pubs Note: papers with two co-first authors are included ONCE in the calculation |
| Group_pubsFIRST_RAonly_publishedfirst_same_type  [numeric] | Mean (sd) : 3.2 (1.3)  min < med < max:  0 < 3 < 9.5  IQR (CV) : 1.7 (0.4) | 86 distinct values | 2122  (92.9%) | 162  (7.1%) | Average of pubs_FIRST_ra_only_publishedfirst for ECRs of the same type from this group in our study (e.g. if type_pre_postdoc = predoc, averages PhD students in this group (postdocs publish faster)) |

Table S2: Overview the current **role in 2021 for PhD and postdoc alumni, with summary classifications of broad career area and classification of the most common (n>25) specific functions within these areas, by cohort**

|  | **PhD Student alumni** | | | | **Postdoc alumni** | | | | **Grand**  **total** |
| --- | --- | --- | --- | --- | --- | --- | --- | --- | --- |
|  | **1997-2004** | **2005-2012** | **2013-2020** | **Total** | **1997-2004** | **2005-2012** | **2013-2020** | **Total** |  |
| **Academic research / teaching / scientific services** | **135 (52.7%)** | **152 (44.6%)** | **198 (53.2%)** | **485 (50.1%)** | **235 (63.7%)** | **237 (56.2%)** | **306 (58.4%)** | **778 (59.2%)** | **1263 (55.3%)** |
| Independent research group leader^1^ (AcPI) | 103 (40.2%) | 90 (26.4%) | 22 (5.9%) | 215 (22.2%) | 176 (47.7%) | 123 (29.1%) | 122 (23.3%) | 421 (32%) | 636 (27.8%) |
| Other academic position^2^ (AcOt) | 29 (11.3%) | 38 (11.1%) | 35 (9.4%) | 102 (10.5%) | 57 (15.4%) | 105 (24.9%) | 119 (22.7%) | 281 (21.4%) | 383 (16.8%) |
| Postdoctoral (AcPD) | 3 (1.2%) | 24 (7%) | 141 (37.9%) | 168 (17.3%) | 2 (0.5%) | 9 (2.1%) | 65 (12.4%) | 76 (5.8%) | 244 (10.7%) |
| **Industry research (IndR)^3^** | **35 (13.7%)** | **54 (15.8%)** | **64 (17.2%)** | **153 (15.8%)** | **38 (10.3%)** | **46 (10.9%)** | **95 (18.1%)** | **179 (13.6%)** | **332 (14.5%)** |
| **Science-related (SciR)** | **44 (17.2%)** | **81 (23.8%)** | **53 (14.2%)** | **178 (18.4%)** | **50 (13.6%)** | **69 (16.4%)** | **52 (9.9%)** | **171 (13%)** | **349 (15.3%)** |
| Administration and training | 6 (2.3%) | 20 (5.9%) | 9 (2.4%) | 35 (3.6%) | 11 (3%) | 14 (3.3%) | 10 (1.9%) | 35 (2.7%) | 70 (3.1%) |
| Business development, consulting, and strategic alliances | 15 (5.9%) | 14 (4.1%) | 9 (2.4%) | 38 (3.9%) | 6 (1.6%) | 9 (2.1%) | 5 (1%) | 20 (1.5%) | 58 (2.5%) |
| Data science, analytics, software engineering^4^ | 1 (0.4%) | 5 (1.5%) | 9 (2.4%) | 15 (1.5%) | 1 (0.3%) | 5 (1.2%) | 7 (1.3%) | 13 (1%) | 28 (1.2%) |
| Intellectual property and law | 6 (2.3%) | 7 (2.1%) | 3 (0.8%) | 16 (1.7%) | 7 (1.9%) | 2 (0.5%) | 1 (0.2%) | 10 (0.8%) | 26 (1.1%) |
| Science writing and communication | 4 (1.6%) | 9 (2.6%) | 3 (0.8%) | 16 (1.7%) | 5 (1.4%) | 8 (1.9%) | 8 (1.5%) | 21 (1.6%) | 37 (1.6%) |
| Technical support and product development | 1 (0.4%) | 11 (3.2%) | 8 (2.2%) | 20 (2.1%) | 3 (0.8%) | 10 (2.4%) | 11 (2.1%) | 24 (1.8%) | 44 (1.9%) |
| All others | 11 (4.3%) | 15 (4.4%) | 12 (3.2%) | 38 (3.9%) | 17 (4.6%) | 21 (5%) | 10 (1.9%) | 48 (3.7%) | 86 (3.8%) |
| **Non-science-related (NonSci)** | **11 (4.3%)** | **19 (5.6%)** | **17 (4.6%)** | **47 (4.9%)** | **13 (3.5%)** | **12 (2.8%)** | **19 (3.6%)** | **44 (3.3%)** | **91 (4%)** |
| Data science, analytics, and software engineering^4^ | 2 (0.8%) | 6 (1.8%) | 10 (2.7%) | 18 (1.9%) | 7 (1.9%) | 7 (1.7%) | 11 (2.1%) | 25 (1.9%) | 43 (1.9%) |
| All others | 9 (3.5%) | 13 (3.8%) | 7 (1.9%) | 29 (3%) | 6 (1.6%) | 5 (1.2%) | 8 (1.5%) | 19 (1.4%) | 48 (2.1%) |
| **Unknown** | **31 (12.1%)** | **35 (10.3%)** | **40 (10.8%)** | **106 (10.9%)** | **33 (8.9%)** | **58 (13.7%)** | **52 (9.9%)** | **143 (10.9%)** | **249 (10.9%)** |
| **Total** | 256 | 341 | 372 | 969 | 369 | 422 | 524 | 1315 | 2284 |

Job function classifications are based on a published taxonomy ([Stayart, Brandt, 2020 (1)](#_ENREF_1)). Additional notes:

1) includes those leading an academic research team with financial and scientific independence – evidenced by job title as ‘group leader’, ‘professor’, ‘associate professor’ or ‘tenure-track assistant professor’. Where the status was unclear from the job title, we classified as principal investigators (PIs) if one of the following criteria was fulfilled: a. they appear to directly supervise students/postdocs (based on hierarchy shown on website), b. they have published a last author publication from the current position c. their group website or CV indicates that they have a grant (not just a personal merit fellowship) as a principal investigator. 2) this function differs from the published schema; it includes academic research, scientific services or teaching staff e.g. research staff, teaching faculty and staff, technical directors, research infrastructure engineers 3) this includes alumni carrying out or overseeing scientific research in industry as postdocs,group leaders, research staff, technical directors and research leadership roles, including alumni who appear to be working in computational biology roles of a pharma, biotech, contract research or similar company regardless of job title i.e. including data science roles that appear to be related to analysis of research-related data 4) not including computational biology roles linked to R&D functions

### Table S3: Comparison datasets included in the comparisons in Figure 1C and Figure 1—figure supplement 3

| **Figure** | **Institution** | **Field(s)** | **Source** | **PI-like role defined as** | **Cohorts** | **Timing^^[[1]](#footnote-2)^^** | **Percentage of alumni in PI-like roles** | **#PIs / cohort size** | **Equivalent EMBL data** | **EMBL PIs/**  **cohort**  **size** |
| --- | --- | --- | --- | --- | --- | --- | --- | --- | --- | --- |
| 1-FS3B | University of California, San Francisco | Basic health science | <https://graduate.ucsf.edu/program-statistics>  Data complied 9 Sept 2021 | Tenure-track faculty | 2002-2006  2007-2011  2012-2016 | 10 ys out in 2012-2016  5 yrs out in 2007-2011  1 yr out in 2003-2007  5 yrs out in 2012-2016  1 yr out in 2008-2012  1 yr out in 2013-2017 | 24.7%  7.8%  1.4%  5.9%  1.6%  1.2% | 73/295  23/295  4/295  30/512  8/512  6/521 | 34.3%  16.4%  1.5%  6.9%  1.8%  0.4% | 69/201  33/201  3/201  15/217  4/217  1/246 |
| 1-FS3C | University of Chicago | Bioscience | https://biosciences.uchicago.edu/after-uchicago/outcomes  Data complied 20 Aug 2021 | Faculty incl. nonTT | 2005-2009  2010-2014  2015-2019 | in 2019 (10-14 yrs out)  in 2019 (5-9 yrs out)  in 2019 (0-4 yrs out) | 36.2%  19.2%  5.3% | 113/312  65/339  18/338 | 25.8%  13.8%  2.1% | 56/217  31/225  5/234 |
| 1-FS3D | University of Michigan | Life sciences^^[[2]](#footnote-3)^^ | <https://secure.rackham.umich.edu/academic_information/program_statistics/doctoral/>  Data compiled 9 Nov 2018 | Tenure-track faculty | 2003-2007  2008-2012  2013-2017 | 10 yrs out in 2013-2017  5 yrs out in 2013-2017  1 yr out in 2014-2018 | 26.5%  8.6%  1.4% | 45/170  21/244  4/294 | 28.2%  7.5%  0.4% | 58/206  16/213  1/244 |
| 1-FS3A | Stanford University | Bioscience | <https://irds.stanford.edu/data-findings/phd-jobs>  Data complied 17 Jan 2020 | Tenure-track | 2002-2004  2007-2009 | in 2013 (9-11 yrs out)  in 2013 (4-6 yrs out) | 33.1%  4.7% | 46/139  13/169 | 39.2%  9.6% | 47/120  13/136 |
| 1C | Stanford University | Bioscience | <https://biosciences.stanford.edu/prospective-students/alumni-career-outcomes-dashboard/alumni-career-outcomes-by-cohort/>  Data complied 10 Sept 21^[[3]](#footnote-4)^ | Faculty – research focus | 2000-2005  2006-2010  2011-2015  2016-2019 | in 2018 (13-18 yrs out)  in 2018 (8-12 yrs out)  in 2018 (3-7 yrs out)  in ‘18 or ‘20^^[[4]](#footnote-5)^^ (0-2 yrs out) | 34.0%  26.8%  12.5%  2.8% | 145/426  126/471  63/503  13/472 | 37.1%  22.4%  10.7%  1.6% | 78/210  50/223  25/234  3/185 |
| 1-FS3E | University of Toronto | Life sciences division | Reithmeier, R., et al. (2019). " PLoS One 14(1): e0209898.  <https://www.sgs.utoronto.ca/about/explore-our-data/10000-phds-project/>  Data complied 10 Sep 2021 | Tenure stream | 2000-2003  2004-2007  2008-2011  2012-2015 | in 2016 (13-16 yrs out)  in 2016 (9-12 yrs out)  in 2016 (5-8 yrs out)  in 2016 (1-4 yrs out) | 30.5%  24.9%  18.1%  7.9% | 192/629  203/816  186/1028  97/1234 | 39.4%  28.5%  12.9%  1.6% | 52/132  49/172  22/170  3/193 |

Please note:

- The most recent PhD cohorts and data on PhD destinations 1-year after graduation were not included in the plots in Figure 1C / Figure 1 – Figure Supplement 3. These values are shown in grey. We did not include this data as very few PhD alumni became a PI in the first years after leaving EMBL, consistent with almost all PhDs competing at least one postdoc position before gaining a PI role. A single alumnus/alumna more or less who become PIs at a very early stage will lead to large differences in the relative ratios that does not necessarily reflect a trend to fewer or more PIs in the long-term.
- One published study ([Mathur, Cano, 2018 (2)](#_ENREF_2)) was not included in these comparisons, as a high percentage of alumni (14% of all alumni) were in primarily teaching focused roles – many of these are likely to be included in the faculty numbers. Teaching-focused faculty roles may have different career dynamics to the careers of in research-focussed PI roles. The biomedical focus of the Mathur et al. study may also limit comparability compared to the fundamental life science focus of EMBL.

**Table S4: Hazard ratios of later cohorts compared with earlier reference cohorts, calculated by Cox regression model.**

A hazard ratio lower than 1 indicates decreased likelihood of entering that role.

| **Role of interest** | **Role at EMBL** | **Cohort** | **Reference cohort** | **Hazard ratio** | **95% Confidence Interval** | **p-value** |  | |
| --- | --- | --- | --- | --- | --- | --- | --- | --- |
| **PI** | PhD students | **2005-2012** | **1997-2004** | **0.51** | **0.38 - 0.69** | **1.10E-05** | ******* |  |
|  |  | **2013-2020** | **1997-2004** | **0.23** | **0.14 - 0.38** | **1.90E-08** | ******* |  |
|  |  | 2013-2020 | 2005-2012 | 0.59 | 0.34 - 1.01 | 0.052 |  |  |
|  | Postdocs | **2005-2012** | **1997-2004** | **0.58** | **0.45 - 0.76** | **5.40E-05** | ******* |  |
|  |  | **2013-2020** | **1997-2004** | **0.69** | **0.53 - 0.89** | **0.0054** | ****** |  |
|  |  | 2013-2020 | 2005-2012 | 1.05 | 0.80 - 1.38 | 0.73 |  |  |
| **AcOt** | PhD students | 2005-2012 | 1997-2004 | 1.14 | 0.78 - 1.68 | 0.49 |  |  |
|  |  | 2013-2020 | 1997-2004 | 1.06 | 0.66 - 1.68 | 0.82 |  |  |
|  |  | 2013-2020 | 2005-2012 | 1.03 | 0.67 - 1.59 | 0.89 |  |  |
|  | Postdocs | **2005-2012** | **1997-2004** | **1.56** | **1.16 - 2.10** | **0.0034** | ****** |  |
|  |  | **2013-2020** | **1997-2004** | **1.72** | **1.29 - 2.31** | **0.00027** | ******* |  |
|  |  | 2013-2020 | 2005-2012 | 1.09 | 0.85 - 1.40 | 0.52 |  |  |
| **IndR** | PhD students | 2005-2012 | 1997-2004 | 1.11 | 0.76 - 1.60 | 0.6 |  |  |
|  |  | **2013-2020** | **1997-2004** | **1.60** | **1.08 - 2.36** | **0.018** | ***** |  |
|  |  | **2013-2020** | **2005-2012** | **1.63** | **1.15 - 2.31** | **0.0064** | ****** |  |
|  | Postdocs | 2005-2012 | 1997-2004 | 0.72 | 0.49 - 1.04 | 0.08 |  |  |
|  |  | **2013-2020** | **1997-2004** | **1.51** | **1.09 - 2.10** | **0.013** | ***** |  |
|  |  | **2013-2020** | **2005-2012** | **2.39** | **1.68 - 3.40** | **1.4E-06** | ******* |  |
| **NonRes** | PhD students | **2005-2012** | **1997-2004** | **1.87** | **1.29 - 2.71** | **0.00089** | ******* |  |
|  |  | **2013-2020** | **1997-2004** | **2.33** | **1.47 - 3.71** | **0.00036** | ******* |  |
|  |  | 2013-2020 | 2005-2012 | 1.10 | 0.77 - 1.56 | 0.61 |  |  |
|  | Postdocs | **2005-2012** | **1997-2004** | **1.78** | **1.19 - 2.68** | **0.0055** | ****** |  |
|  |  | **2013-2020** | **1997-2004** | **1.93** | **1.22 - 3.06** | **0.0053** | ****** |  |
|  |  | 2013-2020 | 2005-2012 | 1.05 | 0.73 - 1.51 | 0.79 |  |  |
| **NonSci** | PhD students | **2005-2012** | **1997-2004** | **2.02** | **1.10 - 3.73** | **0.024** | ***** |  |
|  |  | **2013-2020** | **1997-2004** | **2.21** | **1.12 - 4.38** | **0.022** | ***** |  |
|  |  | 2013-2020 | 2005-2012 | 1.06 | 0.63 - 1.76 | 0.84 |  |  |
|  | Postdocs | 2005-2012 | 1997-2004 | 1.44 | 0.67 - 3.08 | 0.35 |  |  |
|  |  | **2013-2020** | **1997-2004** | **2.58** | **1.13 - 5.88** | **0.024** | ***** |  |
|  |  | 2013-2020 | 2005-2012 | 1.63 | 0.84 - 3.18 | 0.15 |  |  |

*<0.05, **<0.01,***<0.001

**Table S5: Hazard ratios for male alumni compared with female alumni, calculated by Cox regression model..**

A hazard ratio lower than 1 indicates decreased likelihood of entering that role.

| **Role of interest** | **Role at EMBL** | **Gender** | **Reference gender** | **Hazard ratio** | **95% Confidence Interval** | **p-value** |  | |
| --- | --- | --- | --- | --- | --- | --- | --- | --- |
| **PI** | PhD students | **male** | **female** | **1.58** | **1.17 - 2.12** | **0.0027** | ****** |  |
|  | Postdocs | **male** | **female** | **1.78** | **1.40 - 2.27** | **2.2e-06** | ******* |  |
| **AcOt** | PhD students | male | female | 1.14 | 0.82 - 1.59 | 0.43 |  |  |
|  | Postdocs | male | female | 1.13 | 0.90 - 1.42 | 0.29 |  |  |
| **IndR** | PhD students | male | female | 1.33 | 0.99 - 1.77 | 0.058 |  |  |
|  | Postdocs | **male** | **female** | **1.60** | **1.19 - 2.15** | **0.002** | ****** |  |
| **NonRes** | PhD students | male | female | 0.81 | 0.61 - 1.08 | 0.15 |  |  |
|  | Postdocs | **male** | **female** | **0.69** | **0.51 - 0.93** | **0.016** | ***** |  |
| **NonSci** | PhD students | **male** | **female** | **1.64** | **1.03 - 2.63** | **0.039** | ***** |  |
|  | Postdocs | **male** | **female** | **4.34** | **1.85 - 10.18** | **0.00075** | ******* |  |

*<0.05, **<0.01,***<0.001

**Table S6: differences in publication statistics by PI status.**

Values that are statistically significant (p<0.05) are highlighted with bold, and those mentioned in paper text are underlined.

|  | **Became PI?** | | | |  | | |
| --- | --- | --- | --- | --- | --- | --- | --- |
| **Publication statistics** | | **no yes** | | **sig~** | | | **Field name** |
| n | | 1599 685 | |  | | |  |
| For: **all publications** from EMBL work | | | | | | | |
| **Number of publications** | | **3.9** | **6.1** | **<2e-16** | | ******* | **PUBS_ALL_TOTAL** |
| **Number of publications with a JIF** | | **3.9** | **6.1** | **<2e-16** | | ******* | **PUBS_ALL_JIF_count** |
| **Mean JIF** | | **9.6** | **10.9** | **1.2E-04** | | ******* | **PUBS_ALL_JIF_mean** |
| **Sum of JIFs** | | **42.1** | **71.9** | **<2e-16** | | ******* | **PUBS_ALL_JIF_SUM** |
| **Highest JIF** | | **17.8** | **24.0** | **<2e-16** | | ******* | **pubs_ALL_JIF_MAX** |
| **Mean of “percentile in subject area”** | | **63.1** | **69.6** | **4.8E-12** | | ******* | **pubs_All_pecentile_mean** |
| **Highest value of “percentile in subject area”** | | **83.9** | **91.7** | **<2e-16** | | ******* | **pubs_All_percentile_MAX** |
| **Mean CNCI** | | **2.6** | **3.1** | **4.6E-02** | | ***** | **PUBS_ALL_CNCI_mean** |
| **Highest value of “CNCI”** | | **8.1** | **11.0** | **2.8E-02** | | ***** | **PUBS_ALL_CNCI_max** |
| **Calendar years: EMBL start year -> earliest publ.** | | **2.5** | **2.1** | **4.5E-11** | | ******* | **pubs_All_published.first** |
| Calendar years: EMBL start year -> last | | 5.4 | 5.5 | 2.0E-01 | |  | pubs_All_published.last |
| **Mean number of authors** | | **9.1** | **7.8** | **1.9E-03** | | ****** | **pubs_All_published_authors_mean** |
| For: **all research articles** from EMBL work | | | | | | | |
| **Number of research articles** | | **3.1** | **4.9** | **<2e-16** | | ******* | **pubs_RAonly_TOTAL** |
| **Number of research articles with a JIF** | | **3.1** | **4.9** | **<2e-16** | | ******* | **pubs_RAonly_JIF_count** |
| **Mean JIF** | | **10.5** | **11.9** | **3.3E-04** | | ******* | **pubs_RAonly_JIF_mean** |
| **Sum of JIFs** | | **38.4** | **62.8** | **8.8E-15** | | ******* | **pubs_RAonly_JIF_SUM** |
| **Highest JIF** | | **17.6** | **23.2** | **2.9E-14** | | ******* | **pubs_RA_JIF_max** |
| **Mean of “percentile in subject area”** | | **71.3** | **77.4** | **6.2E-14** | | ******* | **pubs_RAonly_percentile_mean** |
| **Highest value of “percentile in subject area”** | | **85.2** | **92.2** | **<2e-16** | | ******* | **pubs_RA_pecentile_MAX** |
| **Mean CNCI** | | **2.9** | **3.5** | **1.1E-02** | | ***** | **pubs_RA_CNCI_mean** |
| **Highest value of “CNCI”** | | **6.9** | **10.7** | **2.1E-03** | | ****** | **pubs_RAonly_CNCI_max** |
| **Calendar years: EMBL start-> earliest res.article** | | **2.8** | **2.3** | **7.6E-12** | | ******* | **pubs_RAonly_publishedfirst** |
| Calendar years: EMBL start year -> last | | 5.4 | 5.5 | 3.6E-01 | |  | pubs_RAonly_publishedlast |
| **Mean number of authors** | | **9.7** | **8.2** | **6.5E-04** | | ******* | **pubs_RAonly_Authors_average** |
| For: **first-author research articles** from EMBL work | | | | | | | |
| **Number of 1^st^ author research articles** | | **1.2** | **2.4** | **<2e-16** | | ******* | **pubs_FIRST_ra_only_TOTAL** |
| **Number of 1^st^ author research articles with a JIF** | | **1.2** | **2.4** | **<2e-16** | | ******* | **pubs_FIRST_ra_only_JIF_count** |
| **Mean JIF** | | **9.7** | **12.1** | **7.9E-07** | | ******* | **pubs_FIRST_ra_only_JIF_mean** |
| **Sum of JIFs** | | **16.7** | **30.3** | **<2e-16** | | ******* | **pubs_FIRST_ra_only_JIFsum** |
| **Highest JIF** | | **12.3** | **18.5** | **<2e-16** | | ******* | **pubs_FIRST_RA_JIF_max** |
| **Mean of “percentile in subject area”** | | **69.3** | **77.6** | **7.0E-16** | | ******* | **pubs_FIRST_RA_pecentile_mean** |
| **Highest value of “percentile in subject area”** | | **77.1** | **88.1** | **<2e-16** | | ******* | **pubs_FIRST_RA_pecentile_MAX** |
| **Mean of CNCI** | | **2.2** | **3.3** | **2.6E-04** | | ******* | **pubs_FIRST_RA_CNCI_mean** |
| **Highest value of “CNCI”** | | **3.1** | **5.7** | **2.8E-06** | | ******* | **pubs_FIRST_RA_CNCI_max** |
| **Calendar years: EMBL start -> earliest 1^st^ author** | | **3.6** | **2.8** | **7.5E-15** | | ******* | **pubs_FIRST_ra_only_publishedfirst** |
| Calendar years: EMBL start year -> last | | 4.8 | 4.8 | 9.3E-01 | |  | pubs_FIRST_ra_only_publishedlast |
| **Mean number of authors** | | **7.3** | **6.4** | **6.0E-03** | | ****** | **pubs_FIRST_ra_only_Authors_mean** |
|  | | | | | | | |
| **Number of co-author research articles** | | **1.9** | **2.5** | **1.5E-05** | | ******* | **coauthor_research** |
| **Number of non-research-articles** | | **0.8** | **1.2** | **2.3E-07** | | ******* | **nonresearhc** |

~ statistical significance based on unpaired 2-tailed t-test *<0.05, **<0.01,***<0.001

**Table S7: Hazard ratios of those publishing multiple first author papers compared to those publishing 0 or 1 first author papers calculated by Cox regression model with time from EMBL.**

A hazard ratio lower than 1 indicates decreased likelihood of entering the specified role

| **Entry into** | **Role at EMBL** | **Number of 1st-author** | **Reference # 1st-author** | **Hazard ratio** | **95% Confidence Interval** | **p-value** |  |
| --- | --- | --- | --- | --- | --- | --- | --- |
| PI | PhD | 1 | 0 | 1.73 | 0.92 - 3.25 | 0.092 |  |
|  |  | **2+** | **0** | **4.60** | **2.55 - 8.29** | **4E-07** | ******* |
|  |  | **2+** | **1** | **2.66** | **1.91 - 3.71** | **7.1E-09** | ******* |
|  | postdoc | **1** | **0** | **3.24** | **2.23 - 4.72** | **8.1E-10** | ******* |
|  |  | **2+** | **0** | **6.61** | **4.70 - 9.31** | **2.7E-27** | ******* |
|  |  | **2+** | **1** | **2.04** | **1.59 - 2.60** | **1.4E-08** | ******* |
| AcOt | PhD | 1 | 0 | 1.12 | 0.69 - 1.80 | 0.65 |  |
|  |  | 2+ | 0 | 0.97 | 0.60 - 1.54 | 0.89 |  |
|  |  | 2+ | 1 | 0.87 | 0.61 - 1.24 | 0.45 |  |
|  | postdoc | **1** | **0** | **1.34** | **1.01 - 1.79** | **0.044** | ***** |
|  |  | **2+** | **0** | **1.45** | **1.11 - 1.88** | **0.0062** | ****** |
|  |  | 2+ | 1 | 1.09 | 0.84 - 1.41 | 0.5 |  |
| IndR | PhD | 1 | 0 | 0.91 | 0.61 - 1.36 | 0.65 |  |
|  |  | 2+ | 0 | 0.90 | 0.61 - 1.33 | 0.6 |  |
|  |  | 2+ | 1 | 0.95 | 0.70 - 1.30 | 0.75 |  |
|  | postdoc | **1** | **0** | **0.72** | **0.51 - 1.00** | **0.048** | ***** |
|  |  | **2+** | **0** | **0.65** | **0.48 - 0.88** | **0.0062** | ****** |
|  |  | 2+ | 1 | 0.90 | 0.64 - 1.28 | 0.57 |  |
| NonRes | PhD | **1** | **0** | **0.61** | **0.43 - 0.86** | **0.0052** | ** |
|  |  | **2+** | **0** | **0.37** | **0.25 - 0.53** | **8E-08** | *** |
|  |  | **2+** | **1** | **0.60** | **0.43 - 0.83** | **0.0024** | ** |
|  | postdoc | **1** | **0** | **0.69** | **0.48 - 0.98** | **0.038** | * |
|  |  | **2+** | **0** | **0.37** | **0.26 - 0.55** | **3.5E-07** | *** |
|  |  | **2+** | **1** | **0.54** | **0.36 - 0.82** | **0.0039** | ** |
| NonSci | PhD | 1 | 0 | 0.76 | 0.44 - 1.32 | 0.33 |  |
|  |  | **2+** | **0** | **0.48** | **0.27 - 0.85** | **0.012** | ***** |
|  |  | 2+ | 1 | 0.61 | 0.37 - 1.02 | 0.06 |  |
|  | postdoc | **1** | **0** | **0.36** | **0.17 - 0.77** | **0.0081** | ****** |
|  |  | **2+** | **0** | **0.33** | **0.17 - 0.65** | **0.0014** | ****** |
|  |  | 2+ | 1 | 0.92 | 0.39 - 2.18 | 0.85 |  |

*<0.05, **<0.01,***<0.001

**Table S8: differences in publication statistics by ‘academic research / service / teaching staff’ status.**

Values that are statistically significant (p<0.05) are highlighted with bold, and those mentioned in paper text are underlined.

|  | **Became AcOt?** | | | |  | |
| --- | --- | --- | --- | --- | --- | --- |
| **Publication statistics** | | **no yes** | | **sig~** | | **Field name** |
| Number of alumni | | 1617      667 | |  | |  |
| For: **all publications** from EMBL work | | | | | | |
| **Number of publications** | | **4.4** | **5.0** | **6.9E-03 **** | | **PUBS_ALL_TOTAL** |
| **Number of publications with a JIF** | | **4.4** | **5.0** | **7.6E-03 **** | | **PUBS_ALL_JIF_count** |
| **Mean JIF** | | **10.5** | **9.0** | **2.7E-06 ***** | | **PUBS_ALL_JIF_mean** |
| Sum of JIFs | | 52.4 | 49.5 | 3.1E-01 | | PUBS_ALL_JIF_SUM |
| **Highest JIF** | | **20.4** | **18.4** | **8.0E-03 **** | | **pubs_ALL_JIF_MAX** |
| **Mean of “percentile in subject area”** | | **66.0** | **63.0** | **4.3E-03 **** | | **pubs_All_pecentile_mean** |
| Highest value of “percentile in subject area” | | 86.6 | 86.0 | 5.8E-01 | | pubs_All_percentile_MAX |
| Mean CNCI | | 2.9 | 2.6 | 3.9E-01 | | PUBS_ALL_CNCI_mean |
| Highest value of “CNCI” | | 9.1 | 8.8 | 8.3E-01 | | PUBS_ALL_CNCI_max |
| Calendar years: EMBL start year -> earliest publication | | 2.4 | 2.3 | 3.7E-01 | | pubs_All_published.first |
| Calendar years: EMBL start -> final publication | | 5.4 | 5.5 | 4.8E-01 | | pubs_All_published.last |
| Mean number of authors | | 8.7 | 8.8 | 8.7E-01 | | pubs_All_published_authors_mean |
| For: **all research articles** from EMBL work | | | | | | |
| **Number of research articles** | | **3.5** | **4.0** | **1.4E-02 *** | | **pubs_RAonly_TOTAL** |
| **Number of research articles with a JIF** | | **3.5** | **4.0** | **1.4E-02 *** | | **pubs_RAonly_JIF_count** |
| **Mean JIF** | | **11.4** | **9.9** | **3.7E-05 ***** | | **pubs_RAonly_JIF_mean** |
| Sum of JIFs | | 47.1 | 44.5 | 3.1E-01 | | pubs_RAonly_JIF_SUM |
| **Highest JIF** | | **20.0** | **18.1** | **6.4E-03 **** | | **pubs_RA_JIF_max** |
| **Mean of “percentile in subject area”** | | **73.9** | **72.0** | **4.4E-02 *** | | **pubs_RAonly_percentile_mean** |
| Highest value of “percentile in subject area” | | 87.6 | 87.3 | 7.4E-01 | | pubs_RA_pecentile_MAX |
| Mean CNCI | | 3.1 | 2.9 | 4.1E-01 | | pubs_RA_CNCI_mean |
| Highest value of “CNCI” | | 8.2 | 7.8 | 7.3E-01 | | pubs_RAonly_CNCI_max |
| Calendar years: EMBL start-> earliest res.article articication | | 2.7 | 2.6 | 1.9E-01 | | pubs_RAonly_publishedfirst |
| Calendar years: EMBL start -> final res.article | | 5.4 | 5.4 | 8.6E-01 | | pubs_RAonly_publishedlast |
| Mean number of authors | | 9.1 | 9.3 | 7.5E-01 | | pubs_RAonly_Authors_average |
| For: **first-author research articles** from EMBL work | | | | | | |
| Number of 1^st^ author research articles | | 1.6 | 1.6 | 7.7E-01 | | pubs_FIRST_ra_only_TOTAL |
| No. of 1^st^ author research articles w/ JIF | | 1.6 | 1.6 | 7.7E-01 | | pubs_FIRST_ra_only_JIF_count |
| **Mean JIF** | | **11.2** | **9.2** | **2.7E-05 ***** | | **pubs_FIRST_ra_only_JIF_mean** |
| **Sum of JIFs** | | **22.8** | **19.0** | **5.0E-04 ***** | | **pubs_FIRST_ra_only_JIFsum** |
| **Highest JIF** | | **15.4** | **12.7** | **1.2E-04 ***** | | **pubs_FIRST_RA_JIF_max** |
| **Mean of “percentile in subject area”** | | **73.5** | **69.7** | **1.6E-03 **** | | **pubs_FIRST_RA_pecentile_mean** |
| **Highest value of “percentile in subject area”** | | **82.0** | **79.2** | **1.9E-02 *** | | **pubs_FIRST_RA_pecentile_MAX** |
| Mean of CNCI | | 2.6 | 2.7 | 8.4E-01 | | pubs_FIRST_RA_CNCI_mean |
| Highest value of “CNCI” | | 3.9 | 4.2 | 5.8E-01 | | pubs_FIRST_RA_CNCI_max |
| Calendar years: EMBL start -> earliest 1^st^ author R.A. publication earliest EMBL publication | | 3.3 | 3.2 | 1.8E-01 | | pubs_FIRST_ra_only_publishedfirst |
| Calendar years: EMBL start -> final 1^st^ author | | 4.8 | 4.8 | 8.9E-01 | | pubs_FIRST_ra_only_publishedlast |
| Mean number of authors | | 6.8 | 7.0 | 5.2E-01 | | pubs_FIRST_ra_only_Authors_mean |
|  | | | | | | |
| **Number of co-author research articles** | | **2.0** | **2.4** | **2.8E-03 **** | | **coauthor_research** |
| **Number of publications not classed as research articles (e.g. reviews, conference papers)** | | **0.8** | **1.0** | **3.7E-02 *** | | **nonresearhc** |

~ statistical significance based on unpaired 2-tailed t-test *<0.05, **<0.01,***<0.001

**Table S9: differences in publication statistics by ‘industry research’ status.**

Values that are statistically significant (p<0.05) are highlighted with bold, and those mentioned in paper text are underlined.

|  | **Became IndRes?** | | | |  | | | | |
| --- | --- | --- | --- | --- | --- | --- | --- | --- | --- |
| **Publication statistics** | | **no yes** | | **sig~** | | | **Field name** | | |
| Number of alumni | | 1811 473 | |  | | |  | | |
| For: **all publications** from EMBL work | | | | | | | | | |
| Number of publications | | 4.6 | 4.5 | 7.2E-01 | |  | | PUBS_ALL_TOTAL |  |
| Number of publications with a JIF | | 4.6 | 4.5 | 7.4E-01 | |  | | PUBS_ALL_JIF_count |  |
| Mean JIF | | 10.1 | 9.6 | 1.9E-01 | |  | | PUBS_ALL_JIF_mean |  |
| Sum of JIFs | | 52.3 | 49.0 | 3.3E-01 | |  | | PUBS_ALL_JIF_SUM |  |
| **Highest JIF** | | **20.2** | **18.4** | **2.2E-02** | | ***** | | **pubs_ALL_JIF_MAX** |  |
| Mean of “percentile in subject area” | | 65.2 | 64.8 | 7.4E-01 | |  | | pubs_All_pecentile_mean |  |
| Highest value of “percentile in subject area” | | 86.4 | 86.5 | 9.2E-01 | |  | | pubs_All_percentile_MAX |  |
| Mean CNCI | | 2.8 | 2.6 | 3.9E-01 | |  | | PUBS_ALL_CNCI_mean |  |
| Highest value of “CNCI” | | 9.1 | 8.8 | 8.2E-01 | |  | | PUBS_ALL_CNCI_max |  |
| Calendar years: EMBL start year -> earliest publication | | 2.4 | 2.4 | 5.6E-01 | |  | | pubs_All_published.first |  |
| Calendar years: EMBL start -> final publication | | 5.4 | 5.5 | 9.1E-01 | |  | | pubs_All_published.last |  |
| Mean number of authors | | 8.8 | 8.4 | 3.3E-01 | |  | | pubs_All_published_authors_mean |  |
| For: all **research articles** from EMBL work | | | | | | | | |  |
| Number of research articles | | 3.6 | 3.7 | 7.2E-01 | |  | | pubs_RAonly_TOTAL |  |
| Number of research articles with a JIF | | 3.6 | 3.7 | 6.9E-01 | |  | | pubs_RAonly_JIF_count |  |
| Mean JIF | | 11.1 | 10.4 | 1.2E-01 | |  | | pubs_RAonly_JIF_mean |  |
| Sum of JIFs | | 46.7 | 44.8 | 5.3E-01 | |  | | pubs_RAonly_JIF_SUM |  |
| **Highest JIF** | | **19.9** | **17.9** | **1.1E-02** | | ***** | | **pubs_RA_JIF_max** |  |
| Mean of “percentile in subject area” | | 73.7 | 71.8 | 7.0E-02 | |  | | pubs_RAonly_percentile_mean |  |
| Highest value of “percentile in subject area” | | 87.7 | 86.6 | 2.6E-01 | |  | | pubs_RA_pecentile_MAX |  |
| Mean CNCI | | 3.2 | 2.8 | 1.6E-01 | |  | | pubs_RA_CNCI_mean |  |
| Highest value of “CNCI” | | 8.2 | 7.8 | 7.4E-01 | |  | | pubs_RAonly_CNCI_max |  |
| Calendar years: EMBL start-> earliest res.article articication | | 2.7 | 2.6 | 4.3E-01 | |  | | pubs_RAonly_publishedfirst |  |
| Calendar years: EMBL start -> final res.article | | 5.5 | 5.5 | 9.1E-01 | |  | | pubs_RAonly_publishedlast |  |
| Mean number of authors | | 9.3 | 8.9 | 3.8E-01 | |  | | pubs_RAonly_Authors_average |  |
| For: **first-author research** articles from EMBL work | | | | | | | | |  |
| Number of 1^st^ author research articles | | 1.6 | 1.5 | 2.7E-01 | |  | | pubs_FIRST_ra_only_TOTAL |  |
| Number of 1^st^ author research articles with a JIF | | 1.6 | 1.5 | 3.0E-01 | |  | | pubs_FIRST_ra_only_JIF_count |  |
| Mean JIF | | 10.7 | 10.1 | 3.1E-01 | |  | | pubs_FIRST_ra_only_JIF_mean |  |
| **Sum of JIFs** | | **22.3** | **19.6** | **2.8E-02** | | ***** | | **pubs_FIRST_ra_only_JIFsum** |  |
| **Highest JIF** | | **14.9** | **13.4** | **4.3E-02** | | ***** | | **pubs_FIRST_RA_JIF_max** |  |
| Mean of “percentile in subject area” | | 72.7 | 70.7 | 1.5E-01 | |  | | pubs_FIRST_RA_pecentile_mean |  |
| Highest value of “percentile in subject area” | | 81.4 | 80.0 | 3.2E-01 | |  | | pubs_FIRST_RA_pecentile_MAX |  |
| Mean of CNCI | | 2.7 | 2.4 | 2.1E-01 | |  | | pubs_FIRST_RA_CNCI_mean |  |
| Highest value of “CNCI” | | 4.1 | 3.6 | 1.6E-01 | |  | | pubs_FIRST_RA_CNCI_max |  |
| Calendar years: EMBL start -> earliest 1^st^ author R.A. publication earliest EMBL publication | | 3.3 | 3.3 | 6.0E-01 | |  | | pubs_FIRST_ra_only_publishedfirst |  |
| Calendar years: EMBL start -> final 1^st^ author | | 4.8 | 4.8 | 7.8E-01 | |  | | pubs_FIRST_ra_only_publishedlast |  |
| Mean number of authors | | 7.0 | 6.8 | 5.5E-01 | |  | | pubs_FIRST_ra_only_Authors_mean |  |
|  | | | | | | | | |  |
| Number of co-author research articles | | 2.0 | 2.2 | 2.5E-01 | |  | | coauthor_research |  |
| **Number of publications not classed as research articles (e.g. reviews, conference papers)** | | **0.9** | **0.8** | **3.6E-02** | | ***** | | **nonresearhc** |  |

~ statistical significance based on unpaired 2-tailed t-test *<0.05, **<0.01,***<0.001

**Table S10: differences in publication statistics by ‘non-research science-related’ status.**

Values that are statistically significant (p<0.05) are highlighted with bold, and those mentioned in paper text are underlined.

|  | **Became NonRes?** | | | |  | | | |
| --- | --- | --- | --- | --- | --- | --- | --- | --- |
| **Publication statistics** | | **no yes** | | **sig~** | | | **Field name** | |
| Number of alumni | | 1857 427 | |  | | |  | |
| For: **all publications** from EMBL work | | | | | | | | |
| **Number of publications** | | **4.8** | **3.2** | **<2e-16** | | ******* | | **PUBS_ALL_TOTAL** |
| **Number of publications with a JIF** | | **4.8** | **3.2** | **<2e-16** | | ******* | | **PUBS_ALL_JIF_count** |
| Mean JIF | | 10.1 | 9.6 | 2.0E-01 | |  | | PUBS_ALL_JIF_mean |
| **Sum of JIFs** | | **55.2** | **34.9** | **4.3E-15** | | ******* | | **PUBS_ALL_JIF_SUM** |
| **Highest JIF** | | **20.4** | **16.9** | **1.8E-05** | | ******* | | **pubs_ALL_JIF_MAX** |
| Mean of “percentile in subject area” | | 65.1 | 65.3 | 9.1E-01 | |  | | pubs_All_pecentile_mean |
| **Highest value of “percentile in subject area”** | | **87.1** | **83.0** | **9.1E-04** | | ******* | | **pubs_All_percentile_MAX** |
| **Mean CNCI** | | **2.9** | **2.3** | **4.5E-03** | | ****** | | **PUBS_ALL_CNCI_mean** |
| **Highest value of “CNCI”** | | **9.7** | **5.9** | **2.9E-03** | | ****** | | **PUBS_ALL_CNCI_max** |
| **Calendar years: EMBL start year -> earliest publication** | | **2.3** | **2.7** | **5.3E-04** | | ******* | | **pubs_All_published.first** |
| Calendar years: EMBL start -> final publication | | 5.5 | 5.3 | 3.3E-01 | |  | | pubs_All_published.last |
| Mean number of authors | | 8.9 | 8.0 | 5.5E-02 | |  | | pubs_All_published_authors_mean |
| For: **all research articles** from EMBL work | | | | | | | | |
| **Number of research articles** | | **3.9** | **2.6** | **4.1E-16** | | ******* | | **pubs_RAonly_TOTAL** |
| **Number of research articles with a JIF** | | **3.9** | **2.6** | **6.4E-16** | | ******* | | **pubs_RAonly_JIF_count** |
| Mean JIF | | 11.1 | 10.3 | 7.9E-02 | |  | | pubs_RAonly_JIF_mean |
| **Sum of JIFs** | | **49.5** | **31.4** | **2.0E-14** | | ******* | | **pubs_RAonly_JIF_SUM** |
| **Highest JIF** | | **20.1** | **16.5** | **1.9E-05** | | ******* | | **pubs_RA_JIF_max** |
| Mean of “percentile in subject area” | | 73.6 | 71.8 | 1.1E-01 | |  | | pubs_RAonly_percentile_mean |
| **Highest value of “percentile in subject area”** | | **88.4** | **83.4** | **2.8E-05** | | ******* | | **pubs_RA_pecentile_MAX** |
| **Mean CNCI** | | **3.2** | **2.3** | **4.3E-06** | | ******* | | **pubs_RA_CNCI_mean** |
| **Highest value of “CNCI”** | | **8.9** | **4.4** | **3.7E-09** | | ******* | | **pubs_RAonly_CNCI_max** |
| **Calendar years: EMBL start-> earliest res.article articication** | | **2.6** | **3.0** | **4.2E-04** | | ******* | | **pubs_RAonly_publishedfirst** |
| Calendar years: EMBL start -> final res.article | | 5.5 | 5.4 | 5.5E-01 | |  | | pubs_RAonly_publishedlast |
| **Mean number of authors** | | **9.4** | **8.2** | **8.8E-03** | | ****** | | **pubs_RAonly_Authors_average** |
| For: **first-author research articles** from EMBL work | | | | | | | | |
| **Number of 1^st^ author research articles** | | **1.7** | **1.1** | **2.9E-16** | | ******* | | **pubs_FIRST_ra_only_TOTAL** |
| **Number of 1^st^ author research articles with a JIF** | | **1.7** | **1.1** | **4.3E-16** | | ******* | | **pubs_FIRST_ra_only_JIF_count** |
| **Mean JIF** | | **10.8** | **9.1** | **4.6E-03** | | ****** | | **pubs_FIRST_ra_only_JIF_mean** |
| **Sum of JIFs** | | **23.0** | **15.0** | **2.7E-13** | | ******* | | **pubs_FIRST_ra_only_JIFsum** |
| **Highest JIF** | | **15.2** | **11.5** | **2.2E-06** | | ******* | | **pubs_FIRST_RA_JIF_max** |
| Mean of “percentile in subject area” | | 72.8 | 69.9 | 5.4E-02 | |  | | pubs_FIRST_RA_pecentile_mean |
| **Highest value of “percentile in subject area”** | | **81.9** | **77.0** | **7.7E-04** | | ******* | | **pubs_FIRST_RA_pecentile_MAX** |
| **Mean of CNCI** | | **2.7** | **2.0** | **7.4E-04** | | ******* | | **pubs_FIRST_RA_CNCI_mean** |
| **Highest value of “CNCI”** | | **4.3** | **2.7** | **6.2E-05** | | ******* | | **pubs_FIRST_RA_CNCI_max** |
| **Calendar years: EMBL start -> earliest 1^st^ author R.A. publication earliest EMBL publication** | | **3.3** | **3.6** | **1.8E-02** | | ***** | | **pubs_FIRST_ra_only_publishedfirst** |
| Calendar years: EMBL start -> final 1^st^ author | | 4.8 | 4.7 | 2.9E-01 | |  | | pubs_FIRST_ra_only_publishedlast |
| **Mean number of authors** | | **7.2** | **6.1** | **9.6E-05** | | ******* | | **pubs_FIRST_ra_only_Authors_mean** |
|  | | | | | | | | |
| **Number of co-author research articles** | | **2.2** | **1.5** | **3.9E-09** | | ******* | | **coauthor_research** |
| **Number of publications not classed as research articles (e.g. reviews, conference papers)** | | **1.0** | **0.6** | **8.6E-07** | | ******* | | **nonresearhc** |

~ statistical significance based on unpaired 2-tailed t-test *<0.05, **<0.01,***<0.001

**Table S11: differences in publication statistics by ‘non-science role’ status.**

Values that are statistically significant (p<0.05) are highlighted with bold, and those mentioned in paper text are underlined.

|  | **Became NonRes?** | | | |  | | |
| --- | --- | --- | --- | --- | --- | --- | --- |
| **Publication statistics** | | **no yes** | | **sig~** | | | **Field name** |
| Number of alumni | | 2122 162 | |  | | |  |
| For: **all publications** from EMBL work | | | | | | | |
| **Number of publications** | | **4.6** | **3.6** | **2.2E-03** | | ****** | **PUBS_ALL_TOTAL** |
| **Number of publications with a JIF** | | **4.6** | **3.5** | **2.1E-03** | | ****** | **PUBS_ALL_JIF_count** |
| Mean JIF | | 10.1 | 9.2 | 2.9E-01 | |  | PUBS_ALL_JIF_mean |
| **Sum of JIFs** | | **52.4** | **39.4** | **8.3E-03** | | ****** | **PUBS_ALL_JIF_SUM** |
| **Highest JIF** | | **20.0** | **16.8** | **1.7E-02** | | ***** | **pubs_ALL_JIF_MAX** |
| Mean of “percentile in subject area” | | 65.3 | 63.0 | 2.9E-01 | |  | pubs_All_pecentile_mean |
| **Highest value of “percentile in subject area”** | | **86.7** | **81.8** | **1.7E-02** | | ***** | **pubs_All_percentile_MAX** |
| Mean CNCI | | 2.8 | 2.4 | 1.4E-01 | |  | PUBS_ALL_CNCI_mean |
| Highest value of “CNCI” | | 9.2 | 6.9 | 2.8E-01 | |  | PUBS_ALL_CNCI_max |
| Calendar years: EMBL start year -> earliest publication | | 2.4 | 2.4 | 8.8E-01 | |  | pubs_All_published.first |
| Calendar years: EMBL start year -> last | | 5.5 | 5.1 | 5.8E-02 | |  | pubs_All_published.last |
| Mean number of authors | | 8.7 | 9.1 | 6.6E-01 | |  | pubs_All_published_authors_mean |
| For: **all research articles** from EMBL work | | | | | | | |
| **Number of research articles** | | **3.7** | **2.7** | **2.8E-04** | | ******* | **pubs_RAonly_TOTAL** |
| **Number of research articles with a JIF** | | **3.7** | **2.7** | **2.8E-04** | | ******* | **pubs_RAonly_JIF_count** |
| Mean JIF | | 11.1 | 9.9 | 2.0E-01 | |  | pubs_RAonly_JIF_mean |
| **Sum of JIFs** | | **47.1** | **35.0** | **9.6E-03** | | ****** | **pubs_RAonly_JIF_SUM** |
| **Highest JIF** | | **19.6** | **16.4** | **1.9E-02** | | ***** | **pubs_RA_JIF_max** |
| Mean of “percentile in subject area” | | 73.4 | 71.5 | 2.8E-01 | |  | pubs_RAonly_percentile_mean |
| **Highest value of “percentile in subject area”** | | **87.8** | **83.8** | **2.7E-02** | | ***** | **pubs_RA_pecentile_MAX** |
| Mean CNCI | | 3.1 | 2.6 | 1.5E-01 | |  | pubs_RA_CNCI_mean |
| Highest value of “CNCI” | | 8.2 | 6.4 | 3.7E-01 | |  | pubs_RAonly_CNCI_max |
| Calendar years: EMBL start-> earliest res.article articication | | 2.7 | 2.8 | 3.1E-01 | |  | pubs_RAonly_publishedfirst |
| Calendar years: EMBL start -> final res.article | | 5.5 | 5.2 | 1.5E-01 | |  | pubs_RAonly_publishedlast |
| Mean number of authors | | 9.2 | 9.2 | 9.8E-01 | |  | pubs_RAonly_Authors_average |
| For: **first-author research articles** from EMBL work | | | | | | | |
| **Number of 1st author research articles** | | **1.6** | **1.1** | **1.1E-05** | | ******* | **pubs_FIRST_ra_only_TOTAL** |
| **Number of 1st author research articles with a JIF** | | **1.6** | **1.1** | **1.0E-05** | | ******* | **pubs_FIRST_ra_only_JIF_count** |
| **Mean JIF** | | **10.7** | **8.9** | **4.9E-02** | | ***** | **pubs_FIRST_ra_only_JIF_mean** |
| **Sum of JIFs** | | **22.2** | **14.7** | **6.0E-07** | | ******* | **pubs_FIRST_ra_only_JIFsum** |
| **Highest JIF** | | **14.8** | **11.2** | **1.8E-03** | | ****** | **pubs_FIRST_RA_JIF_max** |
| Mean of “percentile in subject area” | | 72.5 | 69.6 | 2.2E-01 | |  | pubs_FIRST_RA_pecentile_mean |
| Highest value of “percentile in subject area” | | 81.4 | 77.0 | 5.7E-02 | |  | pubs_FIRST_RA_pecentile_MAX |
| **Mean of CNCI** | | **2.7** | **1.9** | **2.9E-03** | | ****** | **pubs_FIRST_RA_CNCI_mean** |
| **Highest value of “CNCI”** | | **4.1** | **2.4** | **5.2E-06** | | ******* | **pubs_FIRST_RA_CNCI_max** |
| Calendar years: EMBL start -> earliest 1st author R.A. publication earliest EMBL publication | | 3.3 | 3.5 | 2.9E-01 | |  | pubs_FIRST_ra_only_publishedfirst |
| Calendar years: EMBL start -> final 1^st^ author | | 4.8 | 4.5 | 6.4E-02 | |  | pubs_FIRST_ra_only_publishedlast |
| **Mean number of authors** | | **7.1** | **6.1** | **3.4E-02** | | ***** | **pubs_FIRST_ra_only_Authors_mean** |
|  | | | | | | | |
| **Number of co-author research articles** | | **2.1** | **1.7** | **2.7E-02** | | ***** | **coauthor_research** |
| Number of publications not classed as research articles (e.g. reviews, conference papers) | | 0.9 | 0.8 | 5.4E-01 | |  | nonresearhc |

~ statistical significance based on unpaired 2-tailed t-test *<0.05, **<0.01,***<0.001

**Table S12: differences in publication statistics by cohort.**

Values that are statistically significant (p<0.05) are highlighted with bold, and values mentioned in main text are underlined.

|  |  | | **Cohort** | | | |  | | | | |
| --- | --- | --- | --- | --- | --- | --- | --- | --- | --- | --- | --- |
|  | | 1997 | | 2005- | 20133- |  | |  | |  |  |
| **Publication statistics** | | 2004 | | 2012 | 2020 | **sig~** | | **Field name** | | |  |
| Number of alumni | | 625 | | 763 | 896 |  | | | | | |
| For: **all publications** from EMBL work | | | | | | | | | | | |
| **Number of publications** | | **4.7** | | **4.2** | **4.7** | **2.8E-02** | | ***** | **PUBS_ALL_TOTAL** | | |
| **Number of publications with a JIF** | | **4.7** | | **4.2** | **4.7** | **3.2E-02** | | ***** | **PUBS_ALL_JIF_count** | | |
| **Mean JIF** | | **8.6** | | **9.9** | **11.2** | **2.0E-10** | | ******* | **PUBS_ALL_JIF_mean** | | |
| **Sum of JIFs** | | **45.4** | | **46.3** | **60.3** | **2.4E-06** | | ******* | **PUBS_ALL_JIF_SUM** | | |
| **Highest JIF** | | **18.5** | | **18.6** | **21.7** | **2.4E-05** | | ******* | **pubs_ALL_JIF_MAX** | | |
| Mean of “percentile in subject area” | | 64.6 | | 65.4 | 65.3 | 7.9E-01 | |  | pubs_All_pecentile_mean | | |
| **Highest value of “percentile in subject area”** | | **86.1** | | **84.4** | **88.3** | **1.3E-03** | | ****** | **pubs_All_percentile_MAX** | | |
| **Mean CNCI** | | **2.0** | | **2.3** | **3.7** | **1.2E-09** | | ******* | **PUBS_ALL_CNCI_mean** | | |
| **Highest value of “CNCI”** | | **6.1** | | **6.6** | **13.3** | **6.6E-07** | | ******* | **PUBS_ALL_CNCI_max** | | |
| **Calendar years: EMBL start year -> earliest publication** | | **2.2** | | **2.6** | **2.4** | **1.3E-05** | | ******* | **pubs_All_published.first** | | |
| **Calendar years: EMBL start year -> last publication** | | **5.1** | | **5.7** | **5.5** | **6.5E-05** | | ******* | **pubs_All_published.last** | | |
| **Mean number of authors** | | **5.8** | | **8.0** | **11.3** | **<2e-16** | | ******* | **pubs_All_published_authors_mean** | | |
| For: all **research articles** from EMBL work | | | | | | | | | | | |
| Number of research articles | | 3.8 | | 3.4 | 3.7 | 1.2E-01 | |  | pubs_RAonly_TOTAL | | |
| Number of research articles with a JIF | | 3.8 | | 3.4 | 3.7 | 1.2E-01 | |  | pubs_RAonly_JIF_count | | |
| **Mean JIF** | | **9.4** | | **10.6** | **12.4** | **5.6E-11** | | ******* | **pubs_RAonly_JIF_mean** | | |
| **Sum of JIFs** | | **40.0** | | **41.4** | **54.9** | **2.3E-07** | | ******* | **pubs_RAonly_JIF_SUM** | | |
| **Highest JIF** | | **17.9** | | **18.3** | **21.5** | **3.0E-06** | | ******* | **pubs_RA_JIF_max** | | |
| Mean of “percentile in subject area” | | 72.2 | | 73.1 | 74.2 | 1.5E-01 | |  | pubs_RAonly_percentile_mean | | |
| **Highest value of “percentile in subject area”** | | **87.0** | | **86.2** | **88.9** | **1.5E-02** | | ***** | **pubs_RA_pecentile_MAX** | | |
| **Mean CNCI** | | **2.2** | | **2.6** | **4.1** | **8.7E-11** | | ******* | **pubs_RA_CNCI_mean** | | |
| **Highest value of “CNCI”** | | **5.0** | | **6.3** | **11.8** | **1.3E-07** | | ******* | **pubs_RAonly_CNCI_max** | | |
| **Calendar years: EMBL start -> earliest res.article articication** | | **2.3** | | **2.9** | **2.7** | **4.6E-09** | | ******* | **pubs_RAonly_publishedfirst** | | |
| **Calendar years: EMBL start year -> last res. article** | | **5.1** | | **5.8** | **5.4** | **3.2E-06** | | ******* | **pubs_RAonly_publishedlast** | | |
| **Mean number of authors** | | **5.9** | | **8.3** | **12.2** | **<2e-16** | | ******* | **pubs_RAonly_Authors_average** | | |
| For: **first-author research articles** from EMBL work | | | | | | | | | | | |
| **Number of 1^st^ author research articles** | | **1.9** | | **1.5** | **1.4** | **1.3E-10** | | ******* | **pubs_FIRST_ra_only_TOTAL** | | |
| **No. 1^st^ author research articles with JIF** | | **1.9** | | **1.5** | **1.3** | **5.5E-11** | | ******* | **pubs_FIRST_ra_only_JIF_count** | | |
| **Mean JIF** | | **9.2** | | **10.0** | **12.1** | **3.0E-07** | | ******* | **pubs_FIRST_ra_only_JIF_mean** | | |
| Sum of JIFs | | 22.7 | | 20.3 | 22.3 | 1.6E-01 | |  | pubs_FIRST_ra_only_JIFsum | | |
| **Highest JIF** | | **14.1** | | **13.7** | **15.7** | **1.7E-02** | | ***** | **pubs_FIRST_RA_JIF_max** | | |
| Mean of “percentile in subject area” | | 71.8 | | 72.3 | 72.7 | 8.0E-01 | |  | pubs_FIRST_RA_pecentile_mean | | |
| Highest value of “percentile in subject area” | | 82.0 | | 80.3 | 81.2 | 4.4E-01 | |  | pubs_FIRST_RA_pecentile_MAX | | |
| **Mean of CNCI** | | **2.0** | | **2.5** | **3.1** | **9.6E-04** | | ******* | **pubs_FIRST_RA_CNCI_mean** | | |
| Highest value of “CNCI” | | 3.6 | | 3.9 | 4.5 | 2.3E-01 | |  | pubs_FIRST_RA_CNCI_max | | |
| **Calendar years: EMBL start -> earliest 1^st^ author R.A. publication earliest EMBL publication** | | **2.8** | | **3.5** | **3.5** | **4.9E-12** | | ******* | **pubs_FIRST_ra_only_publishedfirst** | | |
| **Calendar years: EMBL start -> last 1^st^ author R.A. publication earliest EMBL publication** | | **4.5** | | **5.0** | **4.9** | **7.0E-05** | | ******* | **pubs_FIRST_ra_only_publishedlast** | | |
| **Mean number of authors** | | **4.8** | | **6.1** | **9.4** | **<2e-16** | | ******* | **pubs_FIRST_ra_only_Authors_mean** | | |
|  | | | | | | | | | | | |
| **Number of co-author research articles** | | **1.9** | | **1.9** | **2.4** | **5.4E-04** | | ******* | **coauthor_research** | | |
| **Number of publications not classed as research articles (e.g. reviews, conference papers)** | | **0.9** | | **0.8** | **1.0** | **5.5E-03** | | ****** | **nonresearhc** | | |

~ statistical significance based on ANOVA analysis *<0.05, **<0.01,***<0.001

1. Stayart CA, Brandt PD, Brown AM, Dahl T, Layton RL, Petrie KA, et al. Applying inter-rater reliability to improve consistency in classifying PhD career outcomes. F1000Res. 2020;9:8.

2. Mathur A, Cano A, Kohl M, Muthunayake NS, Vaidyanathan P, Wood ME, et al. Visualization of gender, race, citizenship and academic performance in association with career outcomes of 15-year biomedical doctoral alumni at a public research university. PloS one. 2018;13(5):e0197473.

1. Some datasets report career destinations in a single year e.g. 2016, others report destinations at specific timepoints after graduation (e.g. 5 years out from the programme). We include the way that this is reported first e.g. in 2016, or 5-years out – but for clarity, also include the year or timepoint range this corresponds to. [↑](#footnote-ref-2)
2. The statistics from the following programmes, which overlap with EMBL’s research areas, were combined: Bioinformatics, Cellular and Molecular Biology, Microbiology and Immunology, Molecular, Cellular and Developmental Biology, Cancer Biology, Biophysics, Biological Chemistry, Cell and Developmental Biology [↑](#footnote-ref-3)
3. Our analysis was performed with the Stanford data for the career destination in 2018 or 2020 for graduates from 2000-2019; an update for destinations in 2023 for leavers from 2000-2022 has since become available. [↑](#footnote-ref-4)
4. For the 2019 leavers: for this leaving year, the career destination in 2020 (position one calendar year after defence) was used. [↑](#footnote-ref-5)
