# Supplementary material for: The changing career paths of PhDs and postdocs trained at EMBL
Source: eLife. 2023 Nov 23;12:e78706. doi: 10.7554/eLife.78706 (PMC10666930; doi:10.7554/eLife.78706)
Supplement: Supplementary file 2. [file elife-78706-supp2.docx]

**Lu et al. 2023. The changing career paths of PhDs and postdocs trained at EMBL. *eLife* 12:e78706**

### Supplementary File 2: Supplementary Methods

**Identification of alumni for inclusion in the study:**

- Postdocs who spent at least 12-months at EMBL were identified through our alumni records, HR and postdoc office records.
- PhD students who graduated from EMBL’s international PhD programme were identified through our alumni, graduate office and HR records. The thesis defence year was identified using graduate office records.
- Deceased alumni were omitted from the study.

**Basic information**

- Information about gender, nationality and time spent at EMBL was collated from information held in HR records, alumni and graduate and postdoc office records. The data was imported and collated in a password-protected custom-made filemaker database. Information that could be used to easily identify the alumnus were pseudonymized after data compilation (e.g. name, unit & group at EMBL, nationality).
- For PhD students the start year was the start of their PhD and end year was the thesis defence year.
- For postdocs the start and end years were based on the start and end year of their EMBL postdoc contract, including any extensions to that postdoc contract.
- Additional positions at EMBL (e.g. a bridging postdoc contract for former predocs, or a later staff position) were included in the career tracking as a subsequent position, not as a continuation of their time as a PhD student or original postdoc.

**Data collection – career information**

- Career information was identified by carrying out a search for publicly available information (LinkedIN profile, researchgate profile, ORCID record and biosketch or CV on employer or personal website, as well as inclusion on publicly available staff lists). The search engine google was used with the search terms
  - [name]
  - [name] + EMBL OR “European molecular biology laboratory”
  - [name] + last known employer
    - the last known employer was taken from information provided to EMBL’s alumni office. This information was, however, not included unless it could be confirmed with other publicly available information.
- Identity was confirmed only if the publicly available information mentioned having worked/studied at EMBL or if a publicly available listing of their publications was able to confirm the matching identity.
- Basic details of the positions provided in online career information (country, start year and end year) were added to the filemaker database along with a classification of the position (see classification of positions). A calculated field allowed retrieval of the role classification at specific timepoints after EMBL. Where two positions were held in the same calendar year, the most recent position was retrieved.
- Where this was available, we also noted for each postdoc alumni, the year the PhD was awarded.
- If the current position found on personal webpages or profiles appeared to be a temporary academic position (e.g. postdoc or similar) and they had held the position since before 2019, we confirmed this was up-to-date by cross-checking institutional websites. If group pages do not list lab members, we instead checked that there was a publication within the last calendar year with this affiliation.
- Data on positions was initially collected in March 2017-December 2017 for fellows leaving in 2016 or earlier; and updated in May- September 2021 for all fellows. Country names were pseudonymized and employers delinked from the records, after data curation.
- For non-academic roles, we noted whether the job title included one of the following terms ‘manager, leader, senior, head, principal, director, president or chief’; and for non-PI academic roles, we noted whether the role was leading a core facility.

**Classification of positions**

- A detailed three-level classification of roles was carried out based on a published taxonomy <https://f1000research.com/articles/9-8/v2/>. The three-levels of classification include “job sector”, “job type” and “job function”. As it was not always possible to confirm whether academic positions in Europe were research or teaching focussed, whether specific job titles in industry were at the group leader level, and whether roles in industry R&D were computational biology positions, some adaptations were made. These are detailed in the footnotes of Table 1.
- A summary classification into one of six main types of role (independent group leader (AcPI); academic postdoc (AcPD); other academic position (AcOt); industry research (IndR); science related (SciR); and non-science-related (NonSci)) was then calculated based on the detailed classification.
  - AcPI includes those with the job function “Independent research group leader”
  - AcOt includes with the job function “Other academic position”
  - AcPD includes those in the sector academia, with the job function “postdoctoral”
  - IndR includes alumni:
    - with the job function “R&D scientist”
    - with the job function “postdoctoral” and sector “Industry / for-profit”
    - with the career type “research / teaching”, sector “Industry / for-profit”, and function "Business development, consulting, and strategic alliances" OR “Entrepreneurship” (this captures senior leadership roles overseeing industry research strategy, and founders of research- focused start-ups, including small contract research organisations).
  - NonRes includes
    - alumni with job type="Science related non-research”
    - alumni with job type “Research / teaching” who were *not* employed in academia nor had a job function that linked them to industry research [see IndR] (this captures those who are working in teaching outside academia (e.g. secondary education))
  - NonSci includes all remaining positions

**Data collection – publication information**

- Data on all publications linked to EMBL were exported from the Web of Science incites database on 30 June 2021 - including the publication year, research area, type of publication, citation metrics such as percentile in subject area, and journal impact factor for each publication.
  - Data from the main web of science database (WOS) for all EMBL publications was also exported on 30 June 2021, and the full author list and affiliations added by matching the web of science ascension numbers.
  - A small number of publications were listed in WOS but not incites – the article type from WOS was manually added in these cases.
- Alumni in this study were matched to the publications identified from Web of Science as follows:
  - publications during the years ECRs were at EMBL were assigned to individuals without further validation if:
    - the paper was already registered to them in EMBL’s internal publication database
    - there was a match between an author and the individual’s last name and initial, or known variants of their name (e.g. previous surname for alumni who changed their name on marriage) – AND their EMBL supervisor’s name was also on the paper
  - additional publications with matching name & first initial that were published during or after the ECR’s EMBL contract were manually checked and assigned to ECRs if the publication data (full name, affiliation etc) supported that this was a publication from that person and stemmed from EMBL work.
    - an exception was made if the publication was more than 2-calendar years after EMBL and the person was known to have remained or returned to EMBL in a research-based position (e.g. staff scientist) at that time-point.
  - for ECRs with potential name variations (e.g. double last name or special characters), publications were also added manually where appropriate.
- Joint first authorship was established by manually checking all publications where an ECR was the second author. Joint first-authorship contributed to 14.6% of articles classed as first-author. In a small proportion of cases the ECR was the senior (last) author on a research article, and we included this as equivalent to a first-author article [4.8% of articles classed as first-author].
- Once data curation was complete, the publication records were pseudonymized by replacing the web of science ID with a pseudonym, and deletion of additional identifying information not required for the final analysis (e.g. author lists, affiliations, journal name, article title).

**Classification of supervisor**

- The supervisor was classed as senior if, in the year the ECR left, their supervisor was a senior scientist OR had spent >10-years at EMBL.
